# Cellular Signaling Pathways in Insulin Resistance-Systems Biology Analyses of Microarray Dataset Reveals New Drug Target Gene Signatures of Type 2 Diabetes Mellitus

**DOI:** 10.3389/fphys.2017.00013

**Published:** 2017-01-25

**Authors:** Syed Aun Muhammad, Waseem Raza, Thanh Nguyen, Baogang Bai, Xiaogang Wu, Jake Chen

**Affiliations:** ^1^Institute of Molecular Biology and Biotechnology, Bahauddin Zakariya UniversityMultan, Pakistan; ^2^Institute of Biopharmaceutical Informatics and Technologies, Wenzhou Medical UniversityWenzhou, China; ^3^Wenzhou Medical University, 1st Affiliate Hospital WenzhouWenzhou, China; ^4^Department of Computer and Information Science, Purdue UniversityIndianapolis, IN, USA; ^5^Institute for Systems BiologySeattle, WA, USA; ^6^Indiana Center for Systems Biology and Personalized Medicine, Indiana University–Purdue UniversityIndianapolis, IN, USA; ^7^Informatics Institute, School of Medicine, The University of AlabamaBirmingham, AL, USA

**Keywords:** T2DM, microarray dataset, gene signatures, pathways enrichment analysis, drug targets

## Abstract

**Purpose:** Type 2 diabetes mellitus (T2DM) is a chronic and metabolic disorder affecting large set of population of the world. To widen the scope of understanding of genetic causes of this disease, we performed interactive and toxicogenomic based systems biology study to find potential T2DM related genes after cDNA differential analysis.

**Methods:** From the list of 50-differential expressed genes (*p* < 0.05), we found 9-T2DM related genes using extensive data mapping. In our constructed gene-network, T2DM-related differentially expressed seeder genes (9-genes) are found to interact with functionally related gene signatures (31-genes). The genetic interaction network of both T2DM-associated seeder as well as signature genes generally relates well with the disease condition based on toxicogenomic and data curation.

**Results:** These networks showed significant enrichment of insulin signaling, insulin secretion and other T2DM-related pathways including JAK-STAT, MAPK, TGF, Toll-like receptor, p53 and mTOR, adipocytokine, FOXO, PPAR, P13-AKT, and triglyceride metabolic pathways. We found some enriched pathways that are common in different conditions. We recognized 11-signaling pathways as a connecting link between gene signatures in insulin resistance and T2DM. Notably, in the drug-gene network, the interacting genes showed significant overlap with 13-FDA approved and few non-approved drugs. This study demonstrates the value of systems genetics for identifying 18 potential genes associated with T2DM that are probable drug targets.

**Conclusions:** This integrative and network based approaches for finding variants in genomic data expect to accelerate identification of new drug target molecules for different diseases and can speed up drug discovery outcomes.

## Introduction

Type 2 diabetes mellitus (T2DM) is a metabolic and complex disease that is characterized by hyperglycemia in the context of insulin resistance and relative lack of insulin (Kumar et al., 2005). Globally, it is estimated that there are more than 285 million people with T2DM making up about 90% of diabetes cases (Melmed et al., 2011). The disease mechanism is known to a considerable extent and tissues including pancreatic islets, liver, skeletal muscle, adipose tissues, gut, and the immune system play a role in its progression (Kolb and Eizirik, 2011). Although several key factors including lifestyle, diet, obesity and genetic shave been recognized in the progression of insulin resistance and T2DM (Polonsky et al., 1996; Florez, 2008; Ripsin et al., 2009), the underlying mechanisms remain unclear. It has become a progressively challenging health issue due to its high morbidity, mortality, and heightened incidence worldwide (Melmed et al., 2011).

Recent advances revealed that diabetes is a heterogeneous-disease with complex genetic mechanisms. Several biological systems seem to be connected in the progression and development of T2DM; however the limited understanding of the complications of these systems and their interactions has been a major obstruction in the progress of optimal treatments in T2DM. Most cases of diabetes involve many genes, with each being a minor contributor to an intensified possibility of becoming a type 2 diabetic (Melmed et al., 2011) and similarly genes connected with T2DM poorly signify established pathways of insulin signaling (Florez, 2008). The existing methods to find statistically significant functional classes in T2DM related genes have recognized enrichment of cell cycle regulation (McCarthy, 2010; Voight et al., 2010). Nonetheless, the functional categories and therapeutic role of the expressed genes in T2DM and molecular biology of insulin resistance has not been completely understood (Voight et al., 2010). Therefore, significant gaps in clinical outcome still remain within each of these problems, leading investigators to continue searching for more improvement.

Differential expression in islets from diabetic and control individuals explored the list of genes related to type 2 diabetes mellitus. Among the list of probable genes, CHL1, LRFN2, RASGRP1, and PPM1K were significantly associated with insulin secretion and diabetes type 2. During this global expression analysis, it was found that fifty genetic loci associated with T2DM due to genetic co-expression and protein-protein interaction involved in insulin secretion and HbA1c (Taneera et al., 2012). It has been observed that the effect of genetic variations on incessant glycemic events in non-diabetic individuals primarily reveal perturbation of insulin secretion (Jain et al., 2013). Another systems biology approach based on genome wide association studies explored the T2DM pathophysiology and insulin signaling genes (Jain et al., 2013). Similarly, the abnormal secretion of glucagon led to islet inflammation in T2DM and it has been seen the interleukin-6 is involved to stimulate the glucagon secretion (Chow et al., 2014).

Genomic expressions in insulin signaling and integrated pathways may manifest themselves and to interrupt any one of these genes could develop the clinically significant insulin resistance and diabetes (Melmed et al., 2011). The systems biology approach potentially integrate these biological networks and will help in revealing key elements involved in pathogenesis. As genetic expression is vital to better understand the network of systems biology, thereby cDNA microarray technology is a valuable tool for analyzing expression levels of thousands of genes at the same time. The large number of expression datasets in the public domain provides a rich source for genome-wide information on T2DM and affords an opportunity to do expression study with a large number of samples.

**Figure 1:**
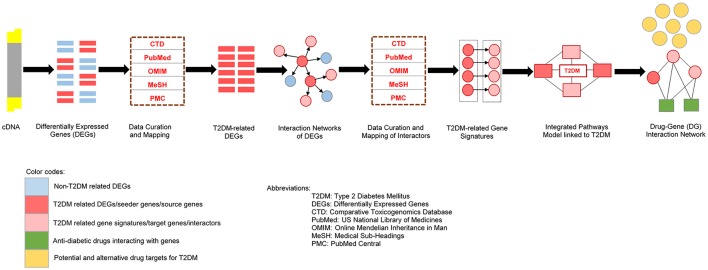
**The comprehensive and squential steps in our study design**.

Therefore, we executed differential analysis to show the target gene signatures associated with insulin resistance and T2DM. In particular, by probing microarray data, we attempted to find a statistically significant T2DM-related differentially expressed genes in diabetic tissue compared to normal. In our study, we constructed the metabolic pathways to uncover new drug targets. We began the analysis by aiming on insulin-signaling and associated cellular genes, a natural and well-established candidates for finding a signature set of genes (Taniquchi et al., 2006) associated with insulin resistance or diabetes. The framework established in this paper is designed to focus key questions: (1) Can biological processes be recognized that are deregulated in metabolic pathways of insulin resistance and diabetes (2) can genetic interaction networks be helpful to reveal new drug targets and biomarkers for optimizing the treatment strategies. Studying these molecular networks from the prospective of probing new drug targets can deliver valuable insights in both biological and medical research. The comprehensive illustration of our study framework has been shown in Figure 1.

## Materials and methods

### Source data

The aim of this study was to find new drug target gene signatures associated with insulin resistance and T2DM. The study design of this dataset indicated to extract RNA from the vastus lateralis of normal (NGT), glucose intolerant (IGT) and type 2 diabetic individuals (total: 118 samples). Our analyses in this study restricted to genes commonly covered by hgu133plus2 chips. We accessed the source expression data for the AffymetrixHG-U133_Plus_2 microarray GSE18732 (Gallagher et al., 2010) from Gene Expression Omnibus database (http://www.ncbi.nlm.nih.gov/geo/query/acc.cgi?acc=GSE18732). The GPL9486 Affymetrix GeneChip Human Genome U133 Plus 2.0 Array (CDF: Hs133P_Hs_ENST, version 10) (Affymetrix, Inc., Santa Clara, CA, 95051, USA, Technology: *in situ* oligonucleotide) platform was used, and the annotation information (hgu133plus2) of probes was used to detect the gene expression. We used computational analysis using R (http://www.r-project.org) and BioConductor (http://www.bioconductor.org) packages.

### Normalization and differential expression analysis

We organized the pheno-data files of this dataset in recognizable format (Troyanskaya et al., 2001). The data was normalized to the median expression level of each gene using the bioconductor “ArrayQuality Metrics” package (Bolstad et al., 2003; Fujita et al., 2006; Obenchain et al., 2014). The expression of a transcript with detection *p*-value 0.15 was considered marginal. We log transformed and quantile normalized the arrays to make sure that they were on the same scale, and computed the gene-gene covariance matrix across all arrays (54675 affyids), ignoring missing values. In order to get a summary of intensities, the Robust Multi-array Analysis (RMA) was used to correct the background (Troyanskaya et al., 2001) for perfect matches (PM) and mismatches (MM). We used the RMA-algorithm to calculate averages between probes in a probe set. To measure the quality of RNA in these samples, AffyRNAdeg, summaryAffyRNAdeg, and plotAffyRNAdeg packages was used for degradation analysis (Affymetrix, 1999, 2001). We performed relative study and identified differentially expressed genes by pair wise comparison from genomic experiments (Tusher et al., 2001) and multiple testing corrections were completed by Benjamini-Hochberg method (Benjamini and Hochberg, 1995). The Limma package, a modified statistic that is proportional to the statistic with sample variance-offsets, was used to shortlist the DEGs and duplicate spots and quality weights were measured. The moderated statistics were calculated; genes were prioritized with respect to the resulting scores and *p*-values. A false discovery rate (FDR) less than 0.05, *p* ≤ 0.05, Average Expression Level (AEL) ≥40% and an absolute log fold change (logFC) greater than 1 were set as the significant cutoffs (Jin and Da, 2013).

### K-Fold validation

We employed K-Fold study of cross-validation and bootstrap for accuracy estimation in differential analysis (Seymour, 1993). The advantage of this method is that all the samples in the dataset are eventually used for both training and testing. K-fold technique is generally better for determining approximate average error and it was used to validate the shortlisted differentially expressed genes using the bioconductor “boot” package. Boots trapping is successfully being used to correct biases in analysis (Ripley, 2010). We applied the generalized linear Gaussian models and used the “cv.glm” function to assess the k-fold cross validation for these cases. The true error is estimated as the average error rate:

(1) $$\begin{array}{l} E=1/K\overset{K}{\underset{i\;=\;K}{\sum }}Ei \end{array}$$

The Gaussian function was trailed by the Leave-One-Out-Cross-Validation (LOOCV) procedure. The LOOCV method is intuitively termed as one is left out as the testing-set and remaining data are used as the training-set (Ripley, 2010). For each experiment, we used N-1 subsets for training and the remaining for testing. The true error is estimated as the average error rate on test cases:

(2) $$\begin{array}{l} E=1/N\overset{N}{\underset{i\;=\;K}{\sum }}Ei \end{array}$$

By increasing the number of folds, the bias of the true error rate estimator will be small and correct (Richard and Dennis, 1984; MAQC Consortium, 2010).

### Disease-gene interaction and cluster analysis

Biomedical text mining system is useful to extract specific information from the literature based on the interactions among different types of biomedical entities (Clematide and Rinaldi, 2012). So, from the list of shortlisted DEGs, we investigated the insulin resistance and T2DM associated genes using diverse data sources including CTD (Comparative Toxicogenomics Database) (http://ctdbase.org/), PubMed (http://www.ncbi.nlm.nih.gov/pubmed), OMIM (Online Mendelian Inheritance in Man) (http://www.ncbi.nlm.nih.gov/omim), MeSH (http://www.ncbi.nlm.nih.gov/mesh) and PMC (http://www.ncbi.nlm.nih.gov/pmc) database to filter disease specific genes.

We performed the Absolute Pearson correlation cluster analysis (Eisen et al., 1998) based on expression values in each sample of T2DM-associated differential expressed genes to explore expression profiling and biological functions (Nam and Kim, 2008) using the CIMminer tool (Scherf et al., 2000).

### Gene network analysis and identifying gene signatures

Proteins usually interact with each other to carry out biological functions (Li et al., 2004; Muhammad et al., 2014) and therefore gene network aims to find biological processes that are steadily deregulated across a cDNA data related with disease conditions in human tissues. In PPI network, each protein is considered as belonging to one or more gene-sets connected with biological or molecular functions (Rachlin et al., 2006). The normal function of these biological networks may show much altered activity in the disease state compared to normal.

To overview the global network of DEGs of microarray dataset, genes in the connection groups were retrieved with a high confidence score (0.999) in the STRING (Search Tool for the Retrieval of Interacting Genes/Proteins) version 10 (Szklarczyk et al., 2011) and HAPPI (Human Annotated and Predicted Protein Interaction) databases (Chen et al., 2009) for protein-protein interactions. These databases mines and annotate comprehensive physical and genetic mapping described in the primary peer-reviewed literature and includes the data that is validated by experimental studies in an inclusive form to support simulation analysis of biological networks and estimation of gene/protein functions. We used Cytoscape software (version 3.2.1) to visualize and analyze molecular and interaction networks (Cline et al., 2007). In this network, we determined the role of each gene signatures (target genes) in type 2 diabetes mellitus that interacted with T2DM-related seeder genes (source genes) by gene mapping using CTD, PubMed, OMIM, MeSH and PMC databases. The motivation for gene-mapping in the network is to find potentially T2DM-related-gene signatures is the hypothesis that genes whose dysfunction contributes to a disease phenotype tend to be functionally related. The total number of gene signatures associated with each seeder protein was measured. We assembled the gene signature that are associated with pathways of interest leading to T2DM and constructed a molecular sub-network of these genes that are highly transcriptionally affected in the diabetes state. We used Network Analyzer in Cytoscape to calculate topological network properties. Nodes in the network were categorized according to the degree of association of gene signature with T2DM. Gene ontology (GO) enrichment of the network help us to show biological functions (Nam and Kim, 2008; Muhammad et al., 2015), and it was carried out using the web-based DAVID (Database for Annotation Visualization and Integrated Discovery) (Huang et al., 2009) and FunRich Annotation tools (Pathan et al., 2015). For these set of gene signatures, *p*-value and FDR were assigned to the number of conditions where it is enriched. The gene-sets with a substantial *p*-value were considered as transcriptionally affected in a wide range of diabetes associated samples.

### Prediction of gene signature specific miRNA targets

MiRNAs are considered as post-transcriptional regulators of a large set of genes involving in many biological processes and signaling pathways. So, a useful step for understanding their functional role is characterizing their influence on the gene targets that help us to understand the disease etiology (Alshalalfa and Alhajj, 2013). Using miRNA influence as a functional signature is promising to find molecular connotations between miRNAs and related gene signatures. MiRNA targets of T2DM-related gene signatures were determined by microRNA target predictor (powered by miRanda, mirSVR) and structure duplex sequences were predicted. MiRNA targets were selected based on the mirSVR score (<=−0.1) which is considered as “good” score (Betel et al., 2008).

### Integrated genome-scale pathway reconstruction with putative T2DM linked genes

A major goal of systems biology is to reconstruct and model *in silico* the metabolic networks of disease related genes. We analyzed the integrated, interactive and metabolic network of T2DM-related gene signatures and observed the correlation between these pathways. Cellular and signaling pathways were reconstructed from the combined gene signatures using PathVisio 3tool (Kutmon et al., 2015). These genes were mapped and curated using KEGG (Kyoto Encyclopedia of Genes and Genomes) pathways on the basis of literature and database evidence. KEGG, a public domain database generally used for gene-enrichment analysis and pathway visualization (Bergholdt et al., 2012; Califano et al., 2012), has a total of 199-unique human pathways with 5197 unique genes/proteins (http://www.genome.jp/kegg/pathway.html). In this integrated network, the potential role of each gene signature in each pathway was studied. To verify known role of these pathways in T2DM, the PubMed was curated using the key words “type and 2 and diabetes and insulin and (signaling or resistance or sensitivity) and (secretion or pancreatic or islets)” in combination with terms indicating each of these pathways. Genes interacted with disease are involved to share functional relationships and represent pathways of interest for the pathogenesis of insulin resistance and T2DM.

### Drug-gene network

In drug-gene network, we investigated for genes that interrelate with anti diabetic-drugs using CTD (http://ctdbase.org/) database. CTD is a source of physically curated chemical-gene, chemical-disease and gene-disease interactions from the literature (Davis et al., 2011). We used chemical-gene interaction query for each gene (T2DM-related) in CTD and accessed drugs using the default parameters. In this interaction, drugs were directly linked with T2DM-associated gene were sorted. We used DrugBank database to verify the FDA-approval status of each drug in the interaction network.

## Results

### Gene expression data and normalization

**Figure 2:**
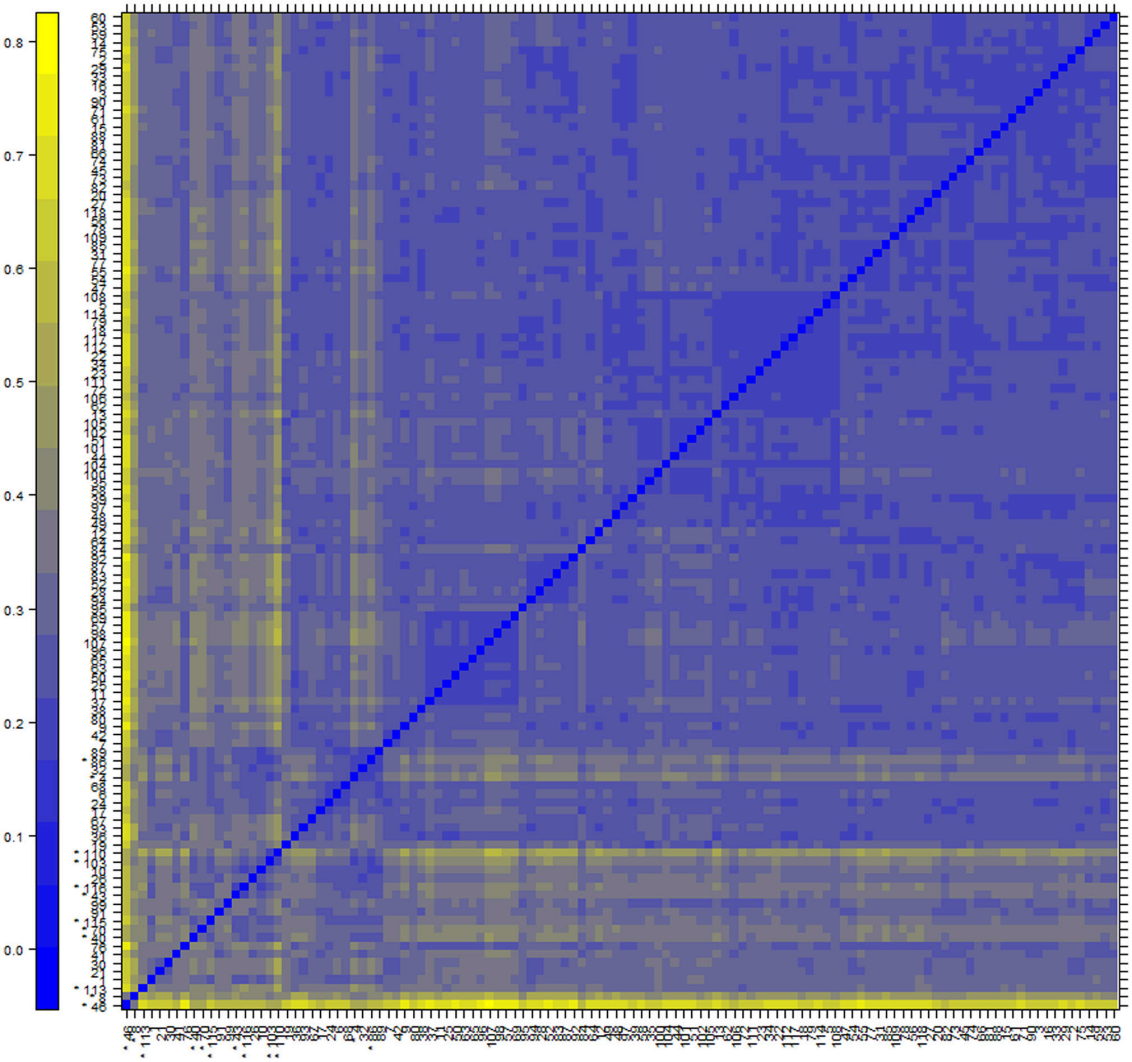
**Normalization and analysis of array quality metrics shows a color heatmap of the distances between arrays**. The color scale is chosen to cover the range of distances encountered in the dataset. Patterns in this plot can indicate clustering of the arrays either because of intended biological or unintended experimental factors (batch effects). The distance d_ab_ between two arrays a and b is computed as the mean absolute difference (L_1_-distance) between the data of the arrays (using the data from all probes without filtering). In formula, d_ab_ = mean | M_ai_ - M_bi_ |, where M_ai_ is the value of the *i*-th probe on the *a*-th array. Outlier detection was performed by looking for arrays for which the sum of the distances to all other arrays, S_*a*_ = Σ_b_ d_ab_ was exceptionally large. 12 such arrays were detected, and they are marked by an asterisk, ^*^.

**Figure 3:**
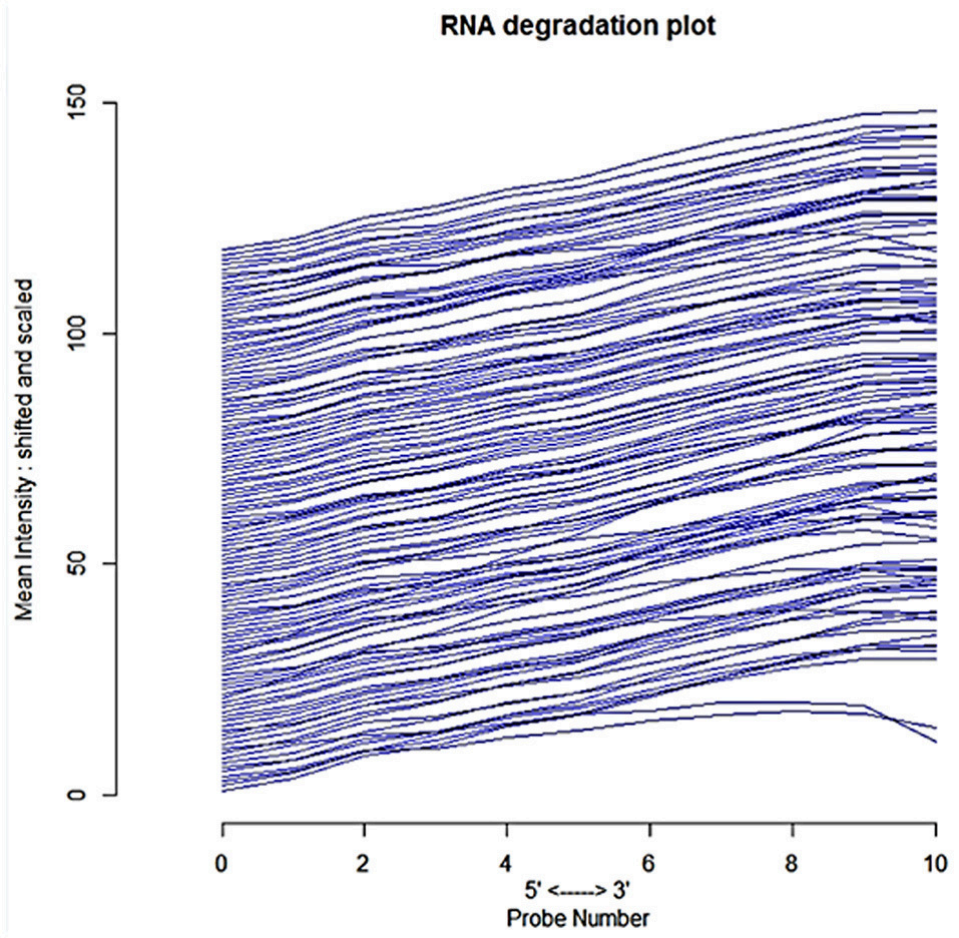
**Side-by-side plot produced by plotAffyRNAdeg representing 5′ to 3′ trendpresenting an assessment of the severity of degradation and significance level**.

We used human GEO dataset to find new drug target gene signatures related to insulin resistance and type 2 diabetes mellitus. The cDNA data has118-samples with 54675 genes derived from the study design of mRNA expression profiling of skeletal muscle of type 2 diabetes (Gallagher et al., 2010). The AffyBatch object comprises the size of the array 1164 × 1164 features with 54675 affyIDS. The quantile normalization of the probes showed quality metrics of the normalized distances between arrays of entire DNA chip. Patterns in this metrics revealed clustering of the arrays either because of intended biological or unintended experimental factors (Figure 2). The individual probes in a probe set was organized by location relative to the 5′-end of the targeted RNA molecule. The 3′/5′ intensity gradient has been shown to depend on the degree of competitive binding of specific and of non-specific targets to a particular probe. Poor RNA quality is related with a reduced amount of RNA quantity hybridized to the array followed by a declined total signal level. Increasing degrees of saturation decrease the 3′/5′ intensity gradient, and we found that short probe sets near the 3′-end of the transcripts (Figure 3). The function summary AffyRNAdeg produced a single summary-statistic for each array in the batch (Supplementary Table 1) indicating an assessment of the severity of RNA-degradation and significance level.

### Identifying differentially expressed genes (DEGs) and cross-validation

An automatic process was used to execute pair-wise comparison between biologically-comparable groups that found a total of 50 DEGs (all down regulated) from expression profiling in the skeletal muscle of normal (NGT), glucose intolerant (IGT) and type 2 diabetic (T2DM) samples (Supplementary Table 2). For reliable results and verification of differential analysis, we let off any sub-group without repetition from the comparisons and the “cv.glm” function of generalized linear models estimated the cross validation prediction error. The dispersion criterion for Gaussian is 0.088314 which shows the confidence level (Table 1). We obtained the same delta value of 0.08847 with K-folds estimation as we used the LOOCV method (during raw cross validation and then during adjusted cross validation). The significant codes (0.1, 0.01, 0.001, and 0.05) with minimum deviance residuals indicated the quality of differential analysis.

**Table 1:** **k-fold cross validation by bioconductor “boot” package using Gaussian dispersion parameters**.

	**Estimate**	**Std. error**	***t*****. value**	**Pr(>|t|)**
(Intercept)	0.038148	0.004451	8.57	<2.00E-16^***^
x1	0.155175	0.005914	26.239	<2.00E-16^***^
x2	−0.03757	0.00669	−5.617	<1.96E-08^***^
x3	0.159384	0.004926	32.357	<2.00E-16^***^
x4	0.157941	0.005739	27.522	<2.00E-16^***^
x5	0.149692	0.005343	28.015	<2.00E-16^***^
x6	0.124573	0.005183	24.034	<2.00E-16^***^
x7	−0.08823	0.002789	−31.636	<2.00E-16^***^
x8	0.130695	0.006101	21.422	<2.00E-16^***^
x9	0.428922	0.004996	85.849	<2.00E-16^***^
x10	0.040628	0.004818	8.433	<2.00E-16^***^
x11	0.132742	0.005614	23.645	<2.00E-16^***^
x12	−0.01105	0.005375	−2.055	0.0399^*^
x13	−0.27454	0.005962	−46.052	<2.00E-16^***^
x14	0.044612	0.005961	7.484	7.29E-14^***^
x15	−0.11979	0.006822	−17.561	<2.00E-16^***^

*Deviance Residuals: Min (−2.6702), 1Q (−0.1516), Median (−0.0100), 3Q (0.1431), Max (4.9980)*.

Signif. codes: 0 ^“***”^ 0.001 ^“**”^ 0.01 ^“*”^ 0.05 “.” 0.1 “” 1.

*Number of Fisher Scoring iterations: 2; $K: [1] 10; $delta: [1] 0.08847 = 0.08846*.

*Null deviance: 171914.5 on 54674 degrees of freedom*.

*Residual deviance: 4827.2 on 54659 degrees of freedom*.

### Identifying T2DM associated genes and cluster analysis

**Figure 4:**
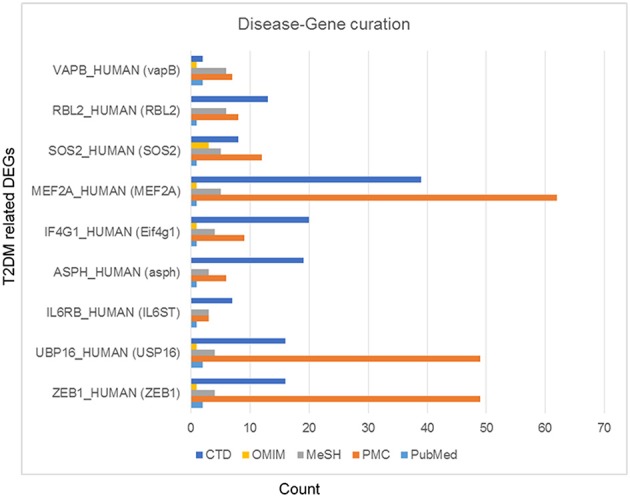
**Type 2 diabetes mellitus specific differentially expressed genes**. These genes were curated using CTD (Comparative Toxicogenomics Database), PubMed, OMIM (Online Mendelian Inheritance in Man), MeSH and PMC databases.

**Figure 5:**
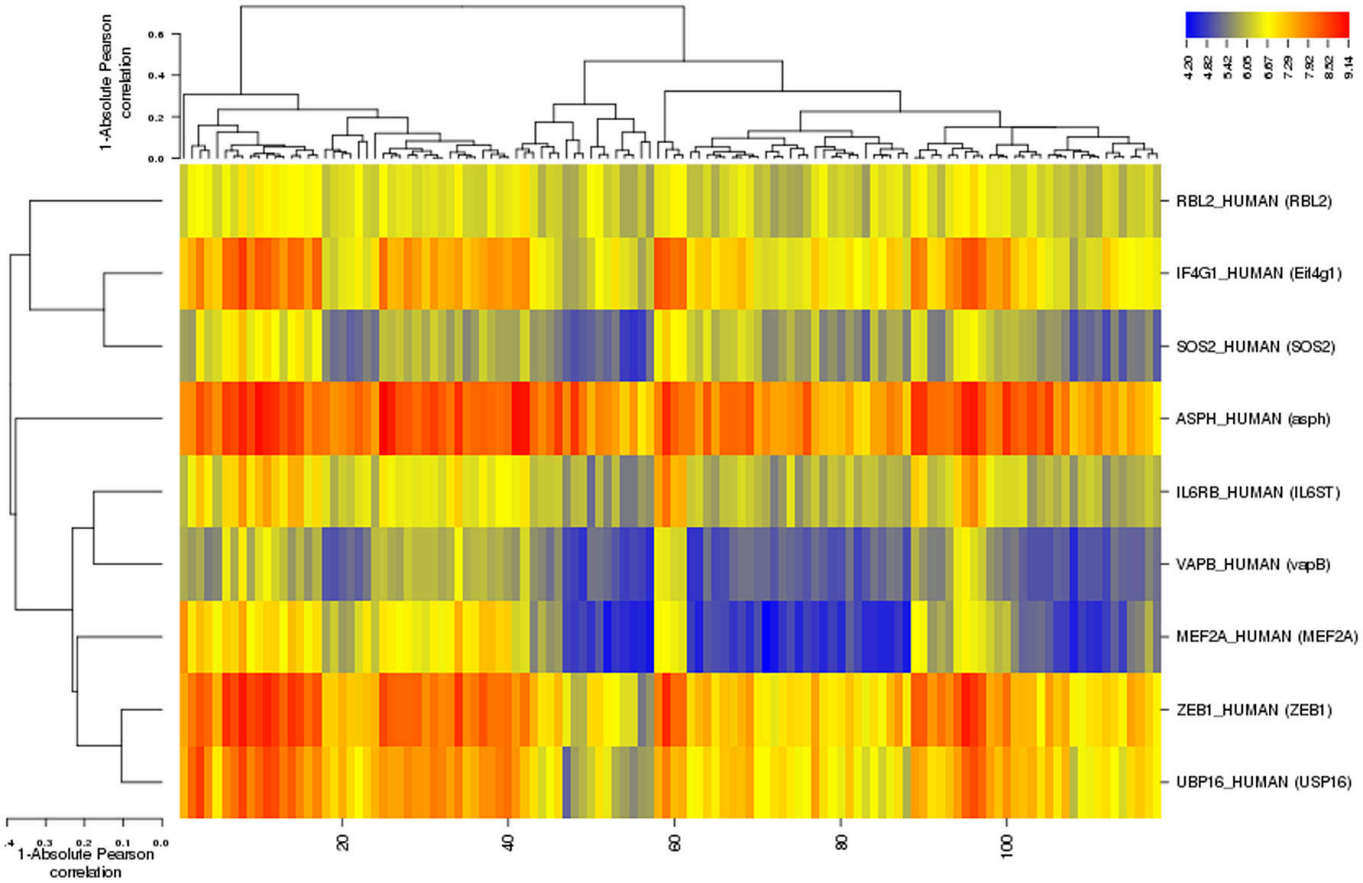
**Cluster analysis of diabetes type 2-related differentialy expressed genes with 1-Absolute Pearson correlation (Binning method: Equal width)**. Blue corresponds to small distance and Red to large distance. Lines indicate the clusters boundaries in the level of the tree.

Among differentially expressed genes, 9 T2DM-related genes were identified including: ZEB1, USP16, IL6ST, ASPH, Eif4g1, RBL2, MEF2A, vapB, and SOS2 after disease-gene interaction using CTD, PubMed, OMIM, MeSH and PMC databases. The role of each gene in T2DM was curated and counted (Figure 4). To show the relationship between these differentially expressed genes and T2DM, we estimated the “similarity” between disease-gene interaction by calculating the Absolute Pearson correlation cluster analysis from two profiles (Figure 5). Clustering analysis has recognized to be helpful to understand gene function, gene regulation, and cellular processes. The genetic expression profiling of skeletal muscle of normal (NGT) is distinguished from the glucose intolerant (IGT) and type 2 diabetic (DM) samples, signifying that obvious differences existed among these cases (treated and untreated).

### Gene network analysis and finding gene signatures

**Figure 6:**
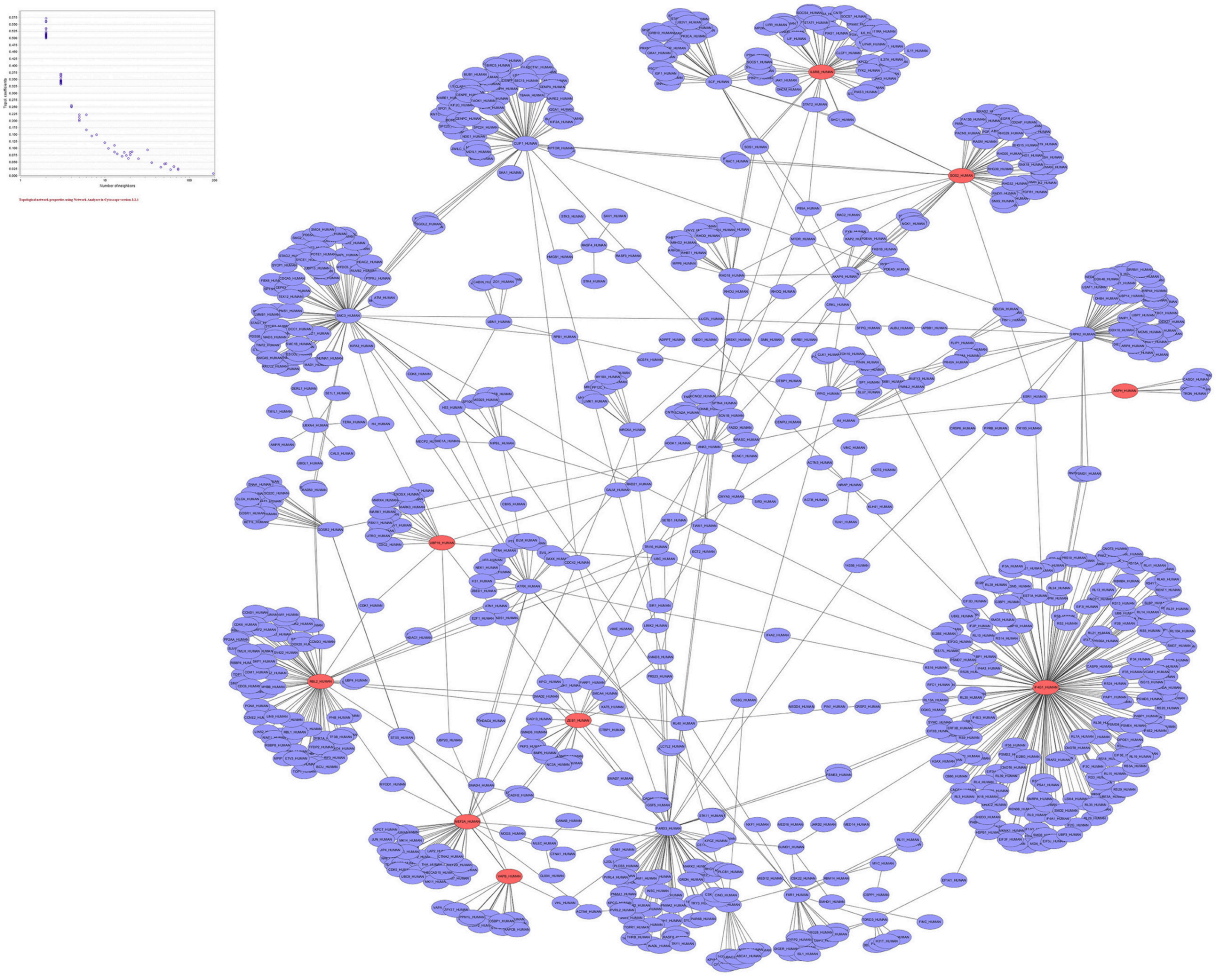
**Genetic network of 50-differentially expressed genes with 885 nodes and 959 edges**. Red nodes representing “T2DM” genes while blue nodes are non-diabetic differentially expressed genes.

**Figure 7:**
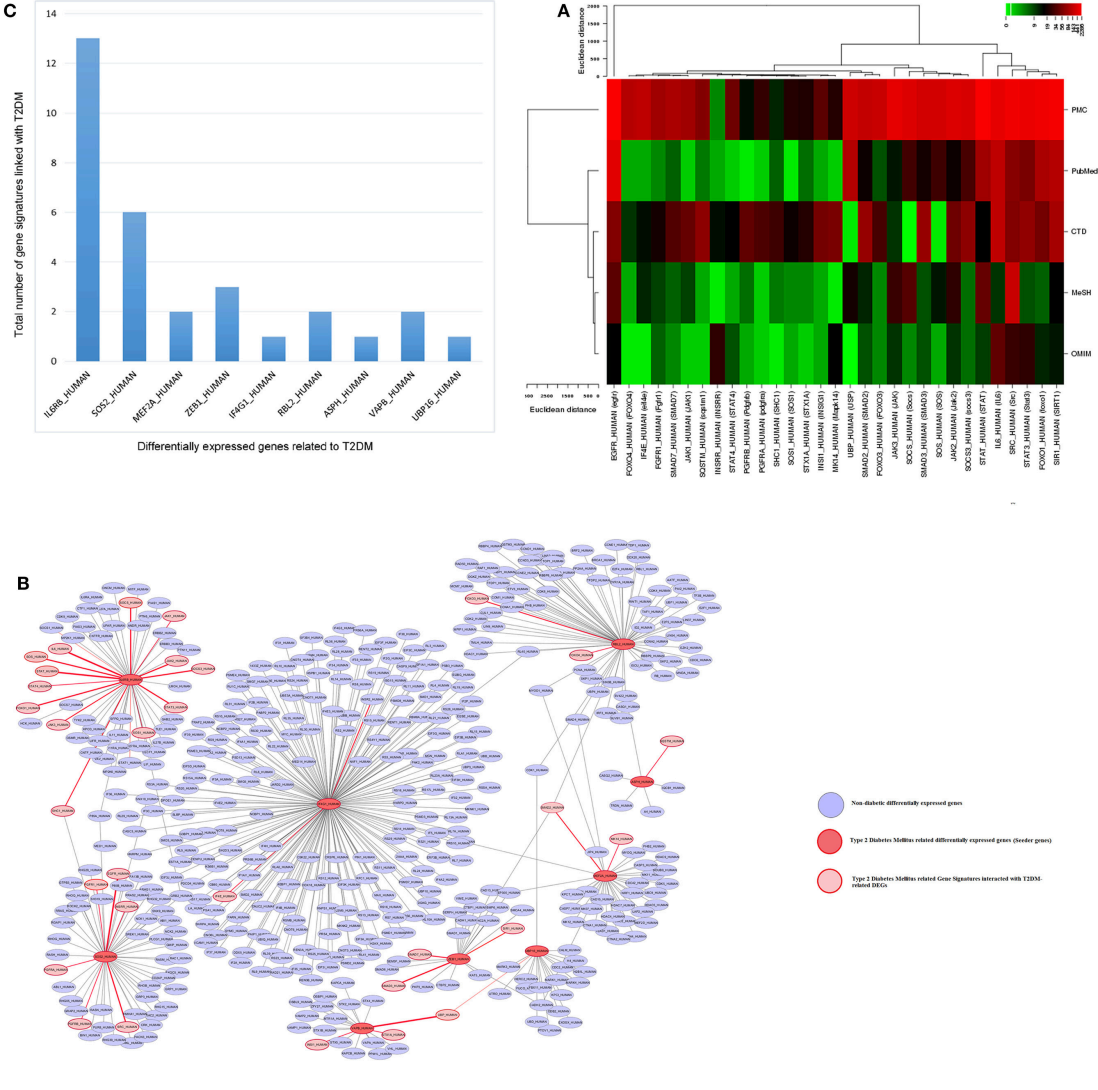
**Molecular Sub-network analysis (A)** gene Mapping and role of gene signatures in T2DM was curated and counted in CTD, PMC, PubMed, OMIM, and MeSH databases **(B)** molecular sub-network (462 nodes and 457 edges) of T2DM-related differentially expressed seeder genes interacted with T2DM-related gene signatures. The interaction is highlighed with red color **(C)** total number of gene signatures associated with each T2DM-related differentially expressed seeder genes.

**Figure 8:**
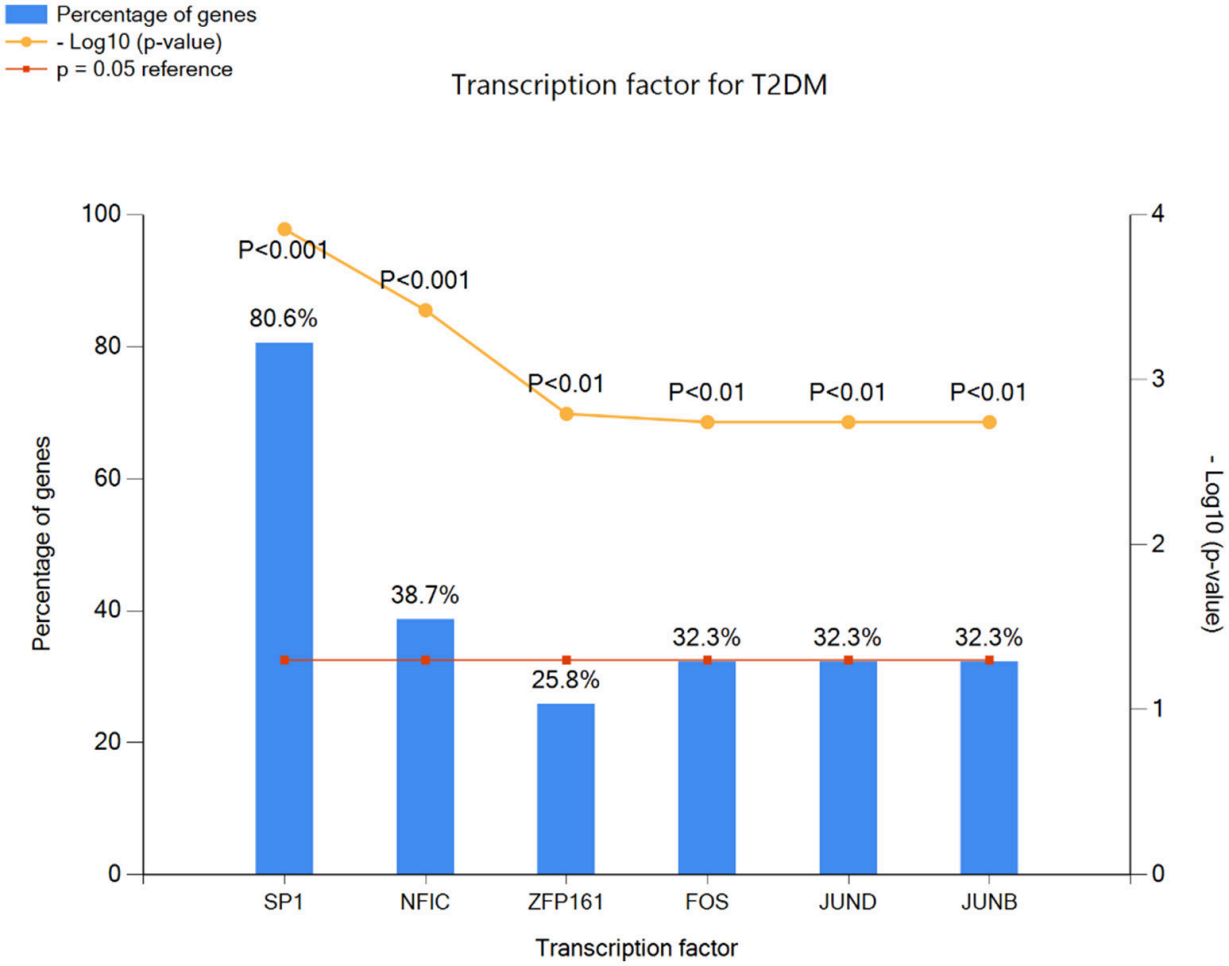
**Transcription factors for T2DM-related gene signatures involved to alter gene expression in a host cell to promote insulin resistance and pathogenesis**.

In genetic network of differentially expressed genes, total of 885 nodes and 959 edges were retrieved from STRING and HAPPI databases (Figure 6). This entire network showed that T2DM-related DEGs were found to interact with other functionally related potential genes that are contributing to a disease phenotype. We identified 31-gene signatures associated with T2DM by disease-gene mapping using CTD, PubMed, OMIM, MeSH and PMC databases (Figure 7A). In the molecular sub-network (462-nodes and 457-edges), these 31-genes were first-order neighbors of the T2DM-related seeder genes. These gene signatures were found to interact with T2DM-related differentially expressed seeder genes: IL6RB_HUMAN (IL6ST), SOS2_HUMAN (SOS2), MEF2A_HUMAN (MEF2A), ZEB1_HUMAN (ZEB1), IF4G1_HUMAN (Eif4g1), RBL2_HUMAN (RBL2), ASPH_HUMAN (ASPH), VAPB_HUMAN (vapB), and UBP16_HUMAN (USP16) (Figure 7B). Using network topology to rank these gene signatures, we identified that among 31-gene signatures, 13 genes had significant connection with IL6RB_HUMAN (IL6ST) seeder gene followed by the 6 with SOS2_HUMAN (SOS2) gene (Figure 7C). In gene ontology (GO) enrichment-based analysis, we selected genes in profile based on fold change combine a *p*-value cut-off (<0.05) which is more consistent selection than those merely based on *p*-value or fold-change alone. These genes are significantly enriched with MAPK cascade, Insulin signaling pathway, interleukin-6-mediated signaling, insulin receptor signaling, JAK-STAT cascade, regulation of insulin secretion and triglyceride metabolic process (Table 2). The significant transcript factors for T2DM were observed including SP1, NFIC, ZFP161, FOS, JUND, and JUNB (Figure 8). We observed transcript abundance in these genes with known T2DM (SP1 80.6%).

**Table 2:** **Gene Ontology and enriched pathways in T2DM-related genes signatures**.

**Term**	***P*****-Value**	**Fold enrichment**	**FDR**
GO:0007167~enzyme linked receptor protein signaling pathway	2.03E-18	21.69175627	3.21E-15
GO:0007169~transmembrane tyrosine kinase signaling pathway	8.32E-13	23.37788018	1.31E-09
GO:0010604~positive regulation of protein metabolic process	5.96E-11	8.147250348	9.41E-08
GO:0042127~regulation of cell proliferation	2.76E-10	8.317416076	4.36E-07
IPR001245:Tyrosine protein kinase	1.30E-09	8.47353586	2.05E-06
GO:0007242~intracellular signaling cascade	7.14E-09	8.505295739	1.13E-05
GO:0007166~cell surface receptor linked signal transduction	3.98E-08	33.88927637	4.41E-05
GO:0016310~phosphorylation	4.08E-08	4.232202447	6.45E-05
GO:0045597~positive regulation of cell differentiation	4.81E-07	9.370036278	5.72E-04
hsa05200:Pathways in cancer	5.77E-07	9.148576452	9.12E-04
GO:0009725~response to hormone stimulus	8.92E-07	64.63373656	0.001027
GO:0007259~JAK-STAT cascade	1.49E-06	18.40418261	0.00171
hsa04630:Jak-STAT signaling pathway	3.01E-06	47.43338008	0.004748
GO:0042981~regulation of apoptosis	3.91E-06	15.56769569	0.004502
GO:0019221~cytokine-mediated signaling pathway	1.45E-05	32.08728653	0.022906
IPR013019:MAD homology, MH1	8.69E-05	5.768124204	0.103221
IPR001132:SMAD domain,	8.72E-05	201.5201613	0.096554
h_egfPathway: EGF Signaling Pathway	6.42E-04	76.37058824	0.7883
GO:0012501~programmed cell death	0.001802	5.074268567	2.809631
hsa04910:Insulin signaling pathway	0.002675	37.74786043	3.133987
GO:0046425~regulation of JAK-STAT cascade	0.002842	7.847222222	2.749384
GO:0031625~ubiquitin protein ligase binding	0.002858	36.3655914	4.421405
GO:0008286~insulin receptor signaling pathway	0.002896	36.06388889	3.511908
h_TPOPathway: TPO Signaling Pathway	0.003845	2.49245367	5.905743
GO:0070102~interleukin-6-mediated signaling pathway	0.006558	293.8390805	7.220592
hsa04350:TGF-beta signaling pathway	0.006687	288.5111111	7.938695
GO:0000165~MAPKKK cascade	0.007536	4.626011627	11.26526
GO:0060397~JAK-STAT in growth hormone signaling pathway	0.008842	218.1935484	13.0922
hsa04062:Chemokine signaling pathway	0.008973	214.9548387	9.502942
IPR013801:STAT transcription factor, DNA-binding	0.012540	153.5391705	13.04699
GO:0005138~interleukin-6 receptor binding	0.015483	15.2228057	21.84943
GO:0007183~SMAD protein complex assembly	0.017556	6.834677419	15.92934
h_il3Pathway:IL 3 signaling pathway	0.020837	2.432792005	22.86529
hsa04920: Adipocytokine signaling pathway	0.036196	5.273560082	44.15
h_aktPathway: AKT Signaling Pathway	0.050395	7.838181818	44.1915
GO:0031016~pancreas development	0.073471	6.480996487	70.05124
GO:0006916~anti-apoptosis	0.075371	6.386152636	71.00653
GO:0042102~positive regulation of T cell proliferation	0.076007	6.355151895	71.32031
GO:0050796~regulation of insulin secretion	0.083053	22.37882548	74.58724
GO:0006641~triglyceride metabolic process	0.087090	5.870678432	76.29865

### Classifying T2DM-gene specific miRNAs targets

MicroRNAs are considered to be important regulators of genes and have already been involved in a growing number of diseases. The computational algorithms (miRanda, mirSVR) predicted T2DM-gene specific multiple miRNA targets including hsa-miR-7, hsa-miR-486-5p, hsa-miR-148b, hsa-miR-140-5p, and hsa-miR-7. The dysregulation of these genes are associated with insulin resistance and type 2 diabetes mellitus. The genes sirt1, pdgfra, shc1, sos, and sos1 predicted 73, 76, 76, 82, and 82 miRNAs hits respectively (Table 3).

**Table 3:** **miRNA targets related to T2DM-related gene signatures**.

**Uniprot_ID**	**Gene_Name**	**microRNA**	**mirSVR score**	**Nuclei mapped to alignments**	**Total MiRNA hits**	**Structure of predicted duplex**
INSI1_HUMAN	INSIG1	hsa-miR-7	−0.1826	170	51	ugUUGUUUUA-GUGA–UCAGAAGGu
INSRR_HUMAN	INSRR	hsa-miR-132	−1.0038	74	5	gcugguaccGACAUCUGACAAu
SOCS_HUMAN	Socs	hsa-miR-324-5p	−0.5384	17	24	ugugguuaCGGGAU–CCCCUACGc
PGFRB_HUMAN	Pdgfrb	hsa-miR-24	−0.3169	217	32	gacaaGGACGACU-UGACUCGGu
STAT_HUMAN	STAT	hsa-miR-421	−0.1519	111	17	cgcgGGUUAA-UUAC—AGACAACUa
EGFR_HUMAN	egfr	hsa-miR-370	−0.1112	46	34	ugGUCCAAGGU-GGGGUCGUCCg
SMAD7_HUMAN	SMAD7	hsa-miR-15b	−1.1638	43	62	acaUUUGGUACUACACGACGAu
SMAD3_HUMAN	SMAD3	hsa-miR-490-3p	−0.1643	1086	19	gucgUACCUC-AGGAGGUCCAAc
UBP_HUMAN	USP	hsa-miR-410	−0.2122	112	18	uguccgguagacacAAUAUAa
JAK3_HUMAN	JAK	hsa-miR-139-5p	−0.1403	143	42	gaccucugUGCACGUGACAUCu
SRC_HUMAN	Src	hsa-miR-491-5p	−0.2503	350	18	ggAGU-ACCUUCCCAAGGGGUGa
UBP16_HUMAN	USP16	hsa-miR-520a-3p	−0.8179	1	27	ugucagguuucccUUCGUGAAa
SOS2_HUMAN	SOS	hsa-miR-148b	−0.2544	45	82	uguuucaagACAUCACGUGACu
JAK2_HUMAN	Jak2	hsa-miR-133a	−0.1218	7	47	gucgaccaacuucccCUGGUUu
SMAD2_HUMAN	SMAD2	hsa-miR-486-5p	−0.1007	288	2	gagcccCGUCGA-GU-CAUGUCCu
STAT3_HUMAN	Stat3	hsa-miR-544	−0.4626	5	47	cuugaacGAUUUUUACGUCUUa
MK14_HUMAN	Mapk14	hsa-miR-421	−0.2044	1	35	cgcGGGUUAAUUAC-AGACAACUa
PGFRA_HUMAN	pdgfra	hsa-miR-140-5p	−0.3169	46	76	gauGGUAUCCCAUUUUGGUGAc
STAT4_HUMAN	STAT4	hsa-miR-132	−1.0501	37	15	gcuggUACCGACAUCUGACAAu
SHC1_HUMAN	SHC1	hsa-miR-140-5p	−0.3169	46	76	gauGGUAUCCCAUUUUGGUGAc
SOS1_HUMAN	SOS1	hsa-miR-148b	−0.2544	45	82	uguuucaagACACUACGUGACu
FOXO4_HUMAN	FOXO4	hsa-miR-149	−0.1391	23	24	cccucacuUCUGUGCCUCGGUCu
SQSTM_HUMAN	sqstm1	hsa-miR-193a-3p	−0.1698	68	36	ugacCCUGAAACAU–CCGGUCAa
FOXO3_HUMAN	FOXO3	hsa-miR-599	−0.1021	34	49	gaugauuuuguacCUUCGUGAAu
SOCS3_HUMAN	socs3	hsa-miR-551a	−0.4552	8	28	acCUUUGGUUCUC–ACCCAGCg
IL6_HUMAN	IL6	hsa-miR-365	−0.1918	16	28	uauucCUAAAAAUCCCCGUAAu
FGFR1_HUMAN	Fgfr1	hsa-miR-133a	−0.1491	241	30	gucgaccaacuuccCCUGGUUu
FOXO1_HUMAN	foxo1	hsa-miR-370	−0.4792	32	60	ugGUCCAAGGUGGGGUCGUCCg
STX1A_HUMAN	STX1A	hsa-miR-491-5p	−0.2032	380	21	ggaguaccuUCCCAAGGGGUGa
IF4E_HUMAN	eif4e	hsa-miR-150	−0.4746	38	62	gugaccauGUUCCCAACCCUCu
JAK1_HUMAN	JAK1	hsa-miR-139-5p	−0.1403	143	42	gaccucugUGCACGUGACAUCu
ZEB1_HUMAN	ZEB1	hsa-miR-217	−0.977	219	62	agGUUAGUCAAGGACUACGUCAu
IL6RB_HUMAN	IL6ST	hsa-miR-873	−0.6274	1	10	uccUCUGAGUGUUCAAGGACg
ASPH_HUMAN	asph	hsa-miR-204	−0.4606	142	52	uccGUAUCCUACUGUUUCCCUu
RBL2_HUMAN	RBL2	hsa-miR-335	−0.1191	1	47	uguaaaaagcaauaacGAGAACu
SIR1_HUMAN	SIRT1	hsa-miR-486-5p	−1.1526	2	73	gagccccguCGAGU-CAUGUCCu

### Pathways model with putative T2DM associated genes

**Figure 9:**
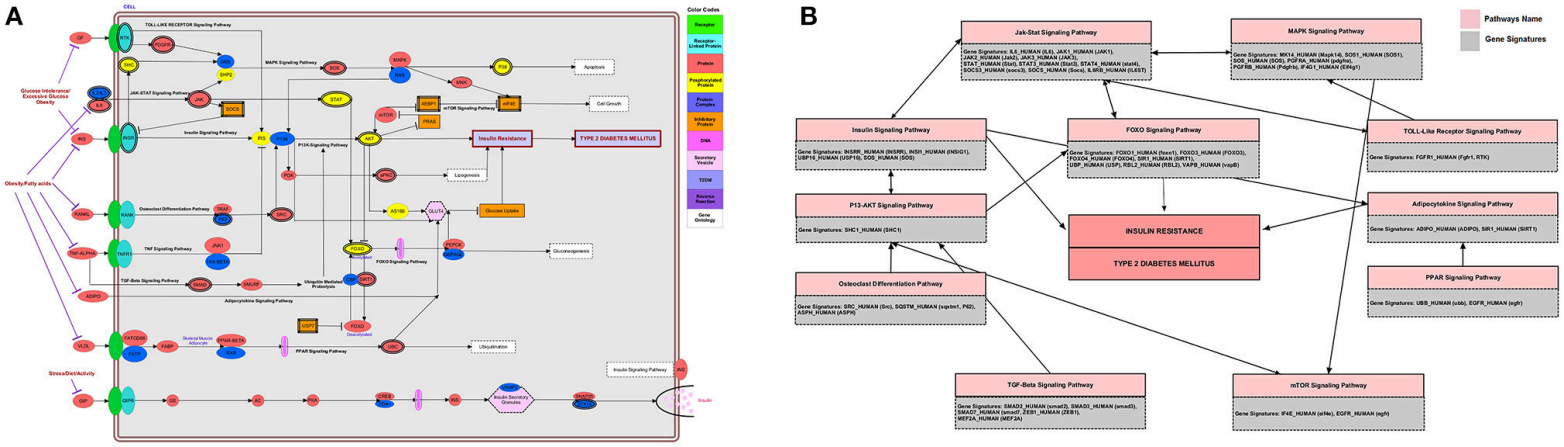
**Pathway analysis (A)** integrated genome to phenome scale signaling pathways involved in insulin resistance and T2DM. Gene signatures were mapped on to KEGG pathway for signaling and metabolic reconstruction **(B)** distribution of T2DM-related gene signatures in associated pathway network.

Genes in T2DM-interactome was studied for pathways modeling which revealed that several pathways are involved in T2DM-pathophysiology. Other than insulin-signaling and T2DM pathway that relates to both insulin secretion and insulin-signaling, the other pathways such as JAK-STAT, MAPK, TGF, Toll-like receptor, p53 and mTOR, adipocytokine, FOXO, PPAR, and P13-AKT signaling pathways have all been connected in T2DM (Figure 9A). Although we found enrichment of several pathways associated with gene signatures, insulin signaling was obvious in over-represented pathways model. Collectively, our analysis determined 11-signaling pathways as a connecting-link between gene signatures in insulin resistance and T2DM. The database was curated to verify the known role of these pathways in T2DM. In this study, we found 8-gene signatures associated with JAK-STAT signaling pathways followed by the FOXO and MAPK pathways (7 and 6-genes respectively) (Figure 9B).

### Finding potential anti-T2DM drug targets in DG-network

**Figure 10:**
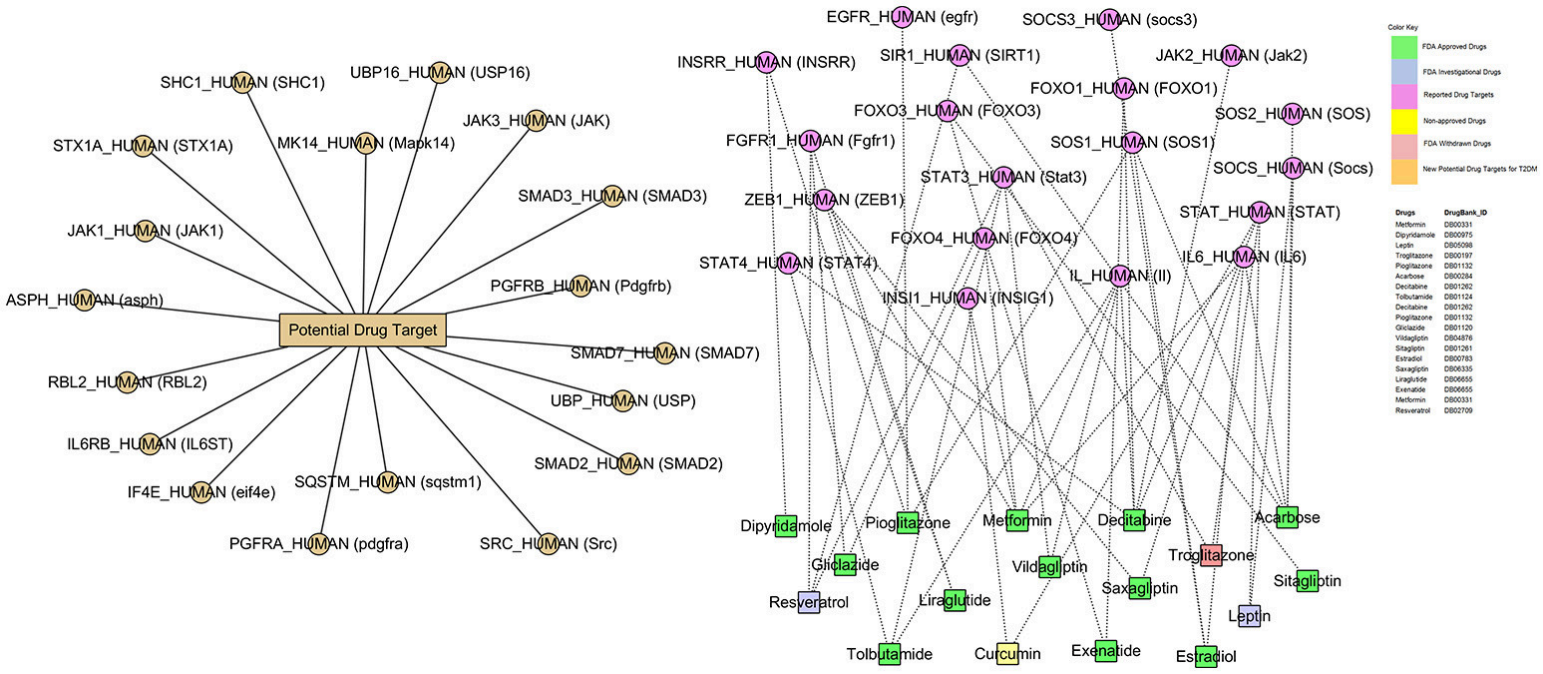
**Drug–Gene network (DG-network)**. The DG-network is generated between the reported drugs and their target gene signatures (55-nodes and 63-edges). Circles and rectangles correspond to target genes and drugs, respectively. A dotted link is placed between a drug and a target node if the gene is a known target of that drug while solid link denotes the potential drug targets. Color codes are given in the legend. DrugBank_ID has been shown for these drugs. The drugs-gene signature assocaition was curated using PMC, CTD, and Drug Bank databases.

We used a toxicogenomic approach for drugs-genes (DG) interaction to further explore the existing treatment and better understanding of disease etiology. Gene that interact with antidiabetic drugs metformin, mipyridamole, leptin, troglitazone, pioglitazone, acarbose, decitabine, tolbutamide, decitabine, gliclazide, vildagliptin, sitagliptin, estradiol, saxagliptin, liraglutide, exenatide, and few others were identified using the publicly available CTD database. Among them, we found 13-FDA approved drugs. In this interaction, we identified 18-genes as potential drug targets (Figure 10) involved in type 2 diabetes mellitus.

## Discussion

The current study signifies the important relationship of genetic variation with gene expression and functional role of these genes in disease. The analyses provide a list of potential T2DM genes based upon differential expression in skeletal muscles, interaction with known T2DM-related gene signatures and correlation with metabolic pathways. The expression profiling of these genes is indicating the obvious differences in skeletal muscle of normal samples from the glucose intolerant (IGT) and type 2 diabetic (DM) samples (Gallagher et al., 2010).

We found 50 down regulated differentially expressed genes that showed the interaction with known T2DM-associated genes (ZEB1, USP16, IL6ST, ASPH, Eif4g1, RBL2, MEF2A, vapB, and SOS2) after mapping in databases. The dysregulation and functional aberration of these differential genes has also been studied (Baxter, 2008; Pihlajamäki et al., 2009; Jewell et al., 2010; Jowett et al., 2010; Nitert et al., 2012; Reddy et al., 2012; Chow et al., 2014; Liew et al., 2014; Neglia et al., 2014) in type 2 diabetes progression. The T2DM linked genes including ZEB1, USP16, IL6ST, ASPH, Eif4g1, RBL2, MEF2A, vapB, and SOS2 effect on pancreatic β-cells, peripheral glucose uptake in muscles, the secretion of multiple cytokines, β-cell gene expression, islet cells, β-cells chromatin and proliferation attenuation (Baxter, 2008; Jowett et al., 2010; Nitert et al., 2012; Chow et al., 2014; Liew et al., 2014). The genetic networks extended the analysis of transcripts to predict interactive gene signatures that have role in diabetes pathophysiology. The molecular sub-network revealed the direct interaction of functionally related 31-gene signatures with seeder genes. In this interaction, we found significant number of gene signatures (13-genes) in connection with T2DM-related IL6ST seeder gene. The variant role of family of IL6-genes has been studied in type 2 diabetes (Chow et al., 2014). Similarly, we observed that SOS2 was another seeder gene that was linked with SRC, INSRR, EGFR, FGFR1, PGFRA and PGFRB gene signatures that were significantly associated with insulin resistance and type 2 diabetes (Davidson et al., 2012; Singh and Kakkar, 2013; Zheng et al., 2013; Li et al., 2015). These observations indicated that the aberration in DEGs expression precede the disturbances in gene signatures ultimately causes type 2 diabetes. The curation and mapping of these gene signatures with T2DM further verified this relationship.

To gain insight into the direction of systems biology, these genes are considerably enriched with Insulin signaling pathway, insulin receptor signaling, interleukin-6-mediated signaling, MAPK cascade, JAK-STAT cascade, regulation of insulin secretion and triglyceride metabolic process. We find that T2DM-gene signatures identified in enrichment analysis can elucidate disease conditions when the interlinked genes are taken together with their protein and functional level interactors (Lee et al., 2011; Lakshmanan et al., 2012; Chow et al., 2014; Ma L. et al., 2015; Ma W. et al., 2015). These signaling pathways contained the significant gene regulatory network of transcript factors families for type 2 diabetes including SP1, NFIC, ZFP161, and FOS, JUND, JUNB. The pathways modeling and integrative network based analysis of gene signatures revealed 11-signaling pathways including insulin secretion, insulin signaling, JAK-STAT, MAPK, TGF, Toll-like receptor, p53 and mTOR, adipocytokine, FOXO, PPAR, and P13-AKT signaling pathways have all been connected in T2DM. Recent reports and literature search indicated our genomic, interactomic, and toxicogenomic evidence to converge on vital pathways including insulin signaling, JAK-STAT signaling, P13-AKT signaling, FOXO signaling, and TGF-beta signaling, the vital pathway involved to play a critical role in pancreatic islets maturity and function, and insulin secretion (Jain et al., 2013). Collectively, we find that genes link directly to insulin secretion and indirectly, through communication with other genes, to insulin resistance and T2DM.

Gene-drug interactions of great interest because such association can not only expressively improve our understanding of disease pathophysiology, but also are helpful in drug discovery processes. The disease related genes-drugs association network can be improved by data mining and biomedical linkages (Chen et al., 2008). Our toxicogenomic-based approach supported this analysis. In this network, 13-FDA approved and few non-approved drugs were associated with T2DM-related genes leaving the 18-genes as potential drug targets. All drugs are FDA approved except troglitazone which has been withdrawn from the market due to its idiosyncratic reaction leading to drug-induced hepatitis. However resveratrol and leptin are FDA investigational drugs and curcumin is non-approved drug. More importantly, this network proposes many testable assumptions with potential of great success, though the real achievement can only be justified by experimental studies.

In conclusion, gene expression microarray studies have greatly improved our knowledge of genetic mechanisms of human diseases. Systems biology analysis of cDNA data helped us to find T2DM-connected genes as alternative drug targets using interactomic and toxicogenomic data that led us to link with vital metabolic and signaling pathways involved in disease pathophysiology. Our simple and integrated steps are helpful in revealing genome to phenome association in diabetes and finding potential drug targets for type 2 diabetes. Therefore this approach will support to understand the genetic basis of complex phenotypes. These findings can provide a valuable framework for developing diagnostic biomarkers and treatment strategies. However, further molecular studies can be designed to validate the role of these genes in T2DM for effective treatment.

## Author contributions

SM and JC designed the study. BB, WR, and XW collected the data. SM, JC, and TN analyzed the data. JC and BB provided guidance with study design and data analysis. All authors contributed to manuscript writing and edition.

### Conflict of interest statement

The authors declare that the research was conducted in the absence of any commercial or financial relationships that could be construed as a potential conflict of interest.
