# Supplementary material for: Cellular Signaling Pathways in Insulin Resistance-Systems Biology Analyses of Microarray Dataset Reveals New Drug Target Gene Signatures of Type 2 Diabetes Mellitus
Source: Front Physiol. 2017 Jan 25;8:13. doi: 10.3389/fphys.2017.00013 (PMC5264126; doi:10.3389/fphys.2017.00013)
Supplement: Supplementary file 1 [file Table1.DOCX]

**Supplementary Table 1**. A summary statistic for each array in the batch, assessing the severity of RNA degradation and significance level

|  | **GSM465274.CEL** | **GSM465275.CEL** | **GSM465276.CEL** | **GSM465277.CEL** | **GSM465278.CEL** | **GSM465279.CEL** | **GSM465280.CEL** | **GSM465281.CEL** |
| --- | --- | --- | --- | --- | --- | --- | --- | --- |
| **slope** | 3.48E+00 | 3.97E+00 | 3.14E+00 | 1.3 | 2.93E+00 | 2.53E+00 | 3.30E+00 | 0.788 |
| **p-value** | 2.47E-12 | 7.66E-13 | 1.57E-11 | 0.000136 | 1.63E-10 | 6.13E-10 | 1.15E-12 | 0.0356 |
|  | **GSM465282.CEL** | **GSM465283.CEL** | **GSM465284.CEL** | **GSM465285.CEL** | **GSM465286.CEL** | **GSM465287.CEL** | **GSM465288.CEL** | **GSM465289.CEL** |
| **slope** | 3.86E+00 | 2.32E+00 | 3.38E+00 | 3.47E+00 | 3.52E+00 | 2.33E+00 | 3.13E+00 | 3.14E+00 |
| **p-value** | 5.97E-13 | 3.96E-09 | 4.99E-12 | 2.19E-12 | 5.41E-12 | 8.79E-10 | 7.35E-11 | 1.74E-11 |
|  | **GSM465290.CEL** | **GSM465291.CEL** | **GSM465292.CEL** | **GSM465293.CEL** | **GSM465294.CEL** | **GSM465295.CEL** | **GSM465296.CEL** | **GSM465297.CEL** |
| **slope** | 2.39E+00 | 3.25E+00 | 3.35E+00 | 2.89E+00 | 4.95E+00 | 2.81E+00 | 2.84E+00 | 2.68E+00 |
| **p-value** | 2.10E-09 | 4.85E-11 | 1.45E-11 | 2.42E-10 | 2.35E-14 | 1.78E-10 | 1.53E-10 | 1.67E-10 |
|  | **GSM465298.CEL** | **GSM465299.CEL** | **GSM465300.CEL** | **GSM465301.CEL** | **GSM465302.CEL** | **GSM465303.CEL** | **GSM465304.CEL** | **GSM465305.CEL** |
| **slope** | 3.18E+00 | 1.39E+00 | 3.54E+00 | 4.24E+00 | 3.42E+00 | 5.55E+00 | 3.10E+00 | 1.65E+00 |
| **p-value** | 4.49E-11 | 8.71E-06 | 1.29E-11 | 2.49E-13 | 2.99E-11 | 3.39E-16 | 8.95E-12 | 1.08E-05 |
|  | **GSM465306.CEL** | **GSM465307.CEL** | **GSM465308.CEL** | **GSM465309.CEL** | **GSM465310.CEL** | **GSM465311.CEL** | **GSM465312.CEL** | **GSM465313.CEL** |
| **slope** | 3.30E+00 | 3.27E+00 | 3.16E+00 | 2.62E+00 | 3.25E+00 | 2.89E+00 | 3.12E+00 | 1.81E+00 |
| **p-value** | 5.12E-12 | 2.37E-11 | 7.83E-11 | 6.64E-10 | 6.83E-12 | 2.09E-13 | 4.21E-11 | 1.33E-07 |
|  | **GSM465314.CEL** | **GSM465315.CEL** | **GSM465316.CEL** | **GSM465317.CEL** | **GSM465318.CEL** | **GSM465319.CEL** | **GSM465320.CEL** | **GSM465321.CEL** |
| **slope** | 1.94E+00 | 3.54E+00 | 3.86E+00 | 3.33E+00 | 2.84E+00 | 1.64E+00 | 2.60E+00 | 3.24E+00 |
| **p-value** | 5.33E-07 | 3.79E-13 | 1.33E-12 | 1.75E-11 | 2.15E-09 | 1.54E-06 | 2.39E-11 | 6.43E-12 |
|  | **GSM465322.CEL** | **GSM465323.CEL** | **GSM465324.CEL** | **GSM465325.CEL** | **GSM465326.CEL** | **GSM465327.CEL** | **GSM465328.CEL** | **GSM465329.CEL** |
| **slope** | 3.68E+00 | 3.55E+00 | 3.51E+00 | 3.81E+00 | 2.36E+00 | 3.00E+00 | 3.30E+00 | 3.28E+00 |
| **p-value** | 5.43E-12 | 3.64E-12 | 3.84E-12 | 4.59E-12 | 1.50E-10 | 1.51E-12 | 4.90E-13 | 1.51E-11 |
|  | **GSM465330.CEL** | **GSM465331.CEL** | **GSM465332.CEL** | **GSM465333.CEL** | **GSM465334.CEL** | **GSM465335.CEL** | **GSM465336.CEL** | **GSM465337.CEL** |
| **slope** | 3.50E+00 | 3.37E+00 | 2.34E+00 | 2.35E+00 | 3.03E+00 | 3.44E+00 | 3.31E+00 | 2.91E+00 |
| **p-value** | 3.82E-12 | 2.89E-11 | 1.34E-09 | 2.16E-10 | 6.30E-11 | 1.07E-11 | 6.33E-12 | 7.62E-12 |
|  | **GSM465338.CEL** | **GSM465339.CEL** | **GSM465340.CEL** | **GSM465341.CEL** | **GSM465342.CEL** | **GSM465343.CEL** | **GSM465344.CEL** | **GSM465345.CEL** |
| **slope** | 3.19E+00 | 3.05E+00 | 3.06E+00 | 2.68E+00 | 3.35E+00 | 3.51E+00 | 3.52E+00 | 2.82E+00 |
| **p-value** | 1.01E-11 | 1.14E-10 | 8.16E-11 | 8.57E-10 | 6.46E-12 | 7.22E-13 | 3.50E-12 | 5.55E-11 |
|  | **GSM465346.CEL** | **GSM465347.CEL** | **GSM465348.CEL** | **GSM465349.CEL** | **GSM465350.CEL** | **GSM465351.CEL** | **GSM465352.CEL** | **GSM465353.CEL** |
| **slope** | 2.87E+00 | 3.11E+00 | 3.73E+00 | 2.95E+00 | 2.76E+00 | 2.95E+00 | 3.45E+00 | 2.91E+00 |
| **p-value** | 4.21E-10 | 7.25E-11 | 1.17E-12 | 1.11E-12 | 1.05E-10 | 3.13E-11 | 1.15E-11 | 2.97E-10 |
|  | **GSM465354.CEL** | **GSM465355.CEL** | **GSM465356.CEL** | **GSM465357.CEL** | **GSM465358.CEL** | **GSM465359.CEL** | **GSM465360.CEL** | **GSM465361.CEL** |
| **slope** | 2.90E+00 | 2.72E+00 | 3.35E+00 | 2.44E+00 | 3.14E+00 | 1.86E+00 | 3.64E+00 | 2.86E+00 |
| **p-value** | 1.29E-10 | 2.25E-09 | 3.47E-12 | 3.82E-10 | 1.10E-11 | 1.26E-07 | 2.32E-12 | 5.22E-10 |
|  | **GSM465362.CEL** | **GSM465363.CEL** | **GSM465364.CEL** | **GSM465365.CEL** | **GSM465366.CEL** | **GSM465367.CEL** | **GSM465368.CEL** | **GSM465369.CEL** |
| **slope** | 2.36E+00 | 3.08E+00 | 1.95E+00 | 3.34E+00 | 4.13E+00 | 4.05E+00 | 3.28E+00 | 2.95E+00 |
| **p-value** | 1.73E-09 | 2.29E-11 | 1.16E-07 | 6.70E-12 | 1.82E-13 | 1.96E-13 | 2.38E-11 | 5.73E-12 |
|  | **GSM465370.CEL** | **GSM465371.CEL** | **GSM465372.CEL** | **GSM465373.CEL** | **GSM465374.CEL** | **GSM465375.CEL** | **GSM465376.CEL** | **GSM465377.CEL** |
| **slope** | 3.48E+00 | 3.57E+00 | 2.88E+00 | 2.82E+00 | 2.95E+00 | 2.91E+00 | 1.51E+00 | 3.43E+00 |
| **p-value** | 5.84E-11 | 5.24E-12 | 1.28E-10 | 4.97E-10 | 8.51E-11 | 1.05E-10 | 9.88E-07 | 3.17E-12 |
|  | **GSM465378.CEL** | **GSM465379.CEL** | **GSM465380.CEL** | **GSM465381.CEL** | **GSM465382.CEL** | **GSM465383.CEL** | **GSM465384.CEL** | **GSM465385.CEL** |
| **slope** | 3.17E+00 | 2.56E+00 | 3.96E+00 | 2.87E+00 | 3.12E+00 | 1.04 | 2.65E+00 | 3.05E+00 |
| **p-value** | 3.06E-11 | 7.32E-10 | 6.19E-13 | 4.53E-10 | 1.21E-11 | 0.00037 | 5.12E-10 | 3.50E-11 |
|  | **GSM465386.CEL** | **GSM465387.CEL** | **GSM465388.CEL** | **GSM465389.CEL** | **GSM465390.CEL** | **GSM465391.CEL** |  |  |
| **slope** | 2.36E+00 | 3.10E+00 | 2.22E+00 | 2.81E+00 | 2.98E+00 | 3.20E+00 |  |  |
| **p-value** | 8.58E-11 | 4.69E-11 | 1.67E-09 | 6.97E-11 | 7.60E-11 | 5.63E-11 |  |  |
