# Supplementary material for: Cellular Signaling Pathways in Insulin Resistance-Systems Biology Analyses of Microarray Dataset Reveals New Drug Target Gene Signatures of Type 2 Diabetes Mellitus
Source: Front Physiol. 2017 Jan 25;8:13. doi: 10.3389/fphys.2017.00013 (PMC5264126; doi:10.3389/fphys.2017.00013)
Supplement: Supplementary file 2 [file Table2.DOCX]

**Supplementary Table 2**. List of 50-differentially expressed genes (all downregulated genes)

| **S. No.** | **Affy_ID** | **Gene_Symbol** | **Uniprot_ID** | **Entereze Gene ID** | **logFC** | **AveExpr** | **t** | **P-Value** | **adj.P.Val** | **B** |
| --- | --- | --- | --- | --- | --- | --- | --- | --- | --- | --- |
| 1 | 203181_x_at | SRPK2 | Q75MW9_HUMAN | 6733 | -0.29149 | 7.194682 | -4.86901 | 3.49E-06 | 0.151069 | 3.39221 |
| 2 | 212007_at | UBXN4 | Q6PJ80_HUMAN | 23190 | -0.77878 | 8.046243 | -4.69489 | 7.21E-06 | 0.151069 | 2.828834 |
| 3 | 212758_s_at | ZEB1 | Q5VZ84_HUMAN | 6935 | -0.77327 | 7.498592 | -4.66105 | 8.29E-06 | 0.151069 | 2.720812 |
| 4 | 1553107_s_at | C5orf24 | CE024_HUMAN | 134553 | -0.30076 | 5.654517 | -4.52898 | 1.42E-05 | 0.1865 | 2.303979 |
| 5 | 209088_s_at | Ubn1 | UBN1_HUMAN | 29855 | -0.56082 | 7.028344 | -4.48321 | 1.71E-05 | 0.1865 | 2.161338 |
| 6 | 200616_s_at | Mlec | MLEC_HUMAN | 9761 | -0.40302 | 7.443705 | -4.41868 | 2.21E-05 | 0.201042 | 1.961855 |
| 7 | 209258_s_at | smc3 | SMC3_HUMAN | 9126 | -0.90516 | 5.242272 | -4.37485 | 2.62E-05 | 0.204969 | 1.827445 |
| 8 | 1570515_a_at | FILIP1 | FLIP1_HUMAN | 27145 | -1.22663 | 6.357146 | -4.30958 | 3.39E-05 | 0.207632 | 1.628927 |
| 9 | 211124_s_at | KITLG | SCF_HUMAN | 4254 | -0.15451 | 3.298804 | -4.29261 | 3.62E-05 | 0.207632 | 1.577661 |
| 10 | 239474_at | slc6a6 | SC6A6_HUMAN | 6533 | -0.19737 | 6.079713 | -4.19197 | 5.35E-05 | 0.207632 | 1.276419 |
| 11 | 1569069_s_at | TDRD3 | TDRD3_HUMAN | 81550 | -0.32753 | 6.705647 | -4.15221 | 6.23E-05 | 0.207632 | 1.158764 |
| 12 | 49306_at | RASSF4 | Q59FL4_HUMAN | 83937 | -0.25018 | 5.547981 | -4.14912 | 6.30E-05 | 0.207632 | 1.149641 |
| 13 | 227954_at | Itpripl2 | IPIL2_HUMAN | 162073 | -0.30274 | 4.276649 | -4.14111 | 6.50E-05 | 0.207632 | 1.126064 |
| 14 | 239571_at | MEF2A | Q7Z6C9_HUMAN | 4205 | -0.94865 | 5.751959 | -4.13774 | 6.58E-05 | 0.207632 | 1.11612 |
| 15 | 221590_s_at | - | - | - | 0.652859 | 5.725371 | 4.13533 | 6.64E-05 | 0.207632 | 1.109042 |
| 16 | 1558924_s_at | CLIP1 | Q6Q318_HUMAN | 6249 | -1.10646 | 7.913786 | -4.12797 | 6.83E-05 | 0.207632 | 1.087419 |
| 17 | 207950_s_at | ANK3 | Q13484_HUMAN | 288 | -0.4318 | 6.774509 | -4.11595 | 7.15E-05 | 0.207632 | 1.052129 |
| 18 | 220072_at | CSPP1 | Q9H688_HUMAN | 79848 | -0.29482 | 5.707059 | -4.11256 | 7.24E-05 | 0.207632 | 1.042195 |
| 19 | 225173_at | arhgap18 | RHG18_HUMAN | 93663 | -0.65617 | 5.594592 | -4.09869 | 7.63E-05 | 0.207632 | 1.001623 |
| 20 | 214464_at | CDC42BPA | Q5T7A7_HUMAN | 8476 | -1.11409 | 5.320669 | -4.09552 | 7.72E-05 | 0.207632 | 0.992366 |
| 21 | 241140_at | Lmo7 | Q5TBK6_HUMAN | 4008 | -0.3304 | 4.822617 | -4.08699 | 7.97E-05 | 0.207632 | 0.967453 |
| 22 | 213206_at | GOSR2 | Q8N4B8_HUMAN | 9570 | 0.217462 | 3.945362 | 4.062763 | 8.74E-05 | 0.208569 | 0.896948 |
| 23 | 222616_s_at | USP16 | UBP16_HUMAN | 10600 | -0.65223 | 7.165454 | -4.06164 | 8.77E-05 | 0.208569 | 0.893694 |
| 24 | 211000_s_at | IL6ST | IL6RB_HUMAN | 3572 | -0.54591 | 6.343641 | -3.99611 | 0.000112 | 0.247246 | 0.704502 |
| 25 | 210094_s_at | Pard3 | Q8IX26_HUMAN | 56288 | -0.28203 | 7.153641 | -3.99364 | 0.000113 | 0.247246 | 0.69741 |
| 26 | 207284_s_at | asph | B4E2K4_HUMAN | 444 | -0.4796 | 7.999178 | -3.98328 | 0.000118 | 0.247246 | 0.667707 |
| 27 | 202549_at | vapB | Q59EZ6_HUMAN | 9217 | -0.48768 | 5.499457 | -3.96026 | 0.000128 | 0.252751 | 0.601927 |
| 28 | 201635_s_at | FXR1 | Q14341_HUMAN | 8087 | -0.53988 | 10.6394 | -3.9494 | 0.000133 | 0.252751 | 0.570986 |
| 29 | 210251_s_at | RUFY3 | RUFY3_HUMAN | 22902 | -0.44593 | 5.657288 | -3.9396 | 0.000138 | 0.252751 | 0.543102 |
| 30 | 235313_at | Nrap | NRAP_HUMAN | 4892 | -1.37894 | 10.28279 | -3.93521 | 0.00014 | 0.252751 | 0.530632 |
| 31 | 242352_at | nipbl | Q6IEH8_HUMAN | 25836 | -0.82858 | 6.153848 | -3.92971 | 0.000143 | 0.252751 | 0.515038 |
| 32 | 208859_s_at | Atrx | Q86U63_HUMAN | 546 | -1.04034 | 5.773837 | -3.91953 | 0.000149 | 0.253517 | 0.486201 |
| 33 | 217576_x_at | SOS2 | SOS2_HUMAN | 6655 | -0.51086 | 5.849823 | -3.91186 | 0.000153 | 0.253517 | 0.464501 |
| 34 | 204341_at | Trim16 | TRI16_HUMAN | 10626 | 0.400223 | 6.806955 | 3.877582 | 0.000173 | 0.27891 | 0.367923 |
| 35 | 1558869_at | AKAP6 | B2RP22_HUMAN | 9472 | -0.20448 | 3.742267 | -3.8626 | 0.000183 | 0.285507 | 0.325912 |
| 36 | 208624_s_at | Eif4g1 | Q4LE58_HUMAN | 1981 | -0.62485 | 7.105241 | -3.85544 | 0.000188 | 0.285507 | 0.30588 |
| 37 | 242665_at | FMNL2 | Q6ZN96_HUMAN | 114793 | -0.31639 | 4.060683 | -3.8245 | 0.00021 | 0.299031 | 0.219593 |
| 38 | 212332_at | RBL2 | Q7Z3L2_HUMAN | 5934 | -0.26205 | 6.334058 | -3.81943 | 0.000214 | 0.299031 | 0.205496 |
| 39 | 235435_at | AASDH | ACSF4_HUMAN | 132949 | -0.5347 | 5.855202 | -3.80176 | 0.000228 | 0.299031 | 0.156519 |
| 40 | 238551_at | FUT11 | FUT11_HUMAN | 170384 | 0.161858 | 4.582903 | 3.801008 | 0.000229 | 0.299031 | 0.154437 |
| 41 | 237981_at | CMYA5 | CMYA5_HUMAN | 202333 | -1.36086 | 10.18963 | -3.79287 | 0.000236 | 0.299031 | 0.131943 |
| 42 | 208994_s_at | PPIG | Q2NKQ6_HUMAN | 9360 | -0.38508 | 6.962639 | -3.79168 | 0.000237 | 0.299031 | 0.12866 |
| 43 | 225381_at | LOC399959 | - | 399959 | 0.364606 | 4.496274 | 3.78857 | 0.000239 | 0.299031 | 0.120057 |
| 44 | 211947_s_at | Bat2l2 | BA2D1_HUMAN | 23215 | -0.55971 | 5.925434 | -3.78702 | 0.000241 | 0.299031 | 0.115768 |
| 45 | 230057_at | LOC285178 | - | 285178 | -0.28733 | 6.141069 | -3.77465 | 0.000252 | 0.301401 | 0.08167 |
| 46 | 241053_at | LOC730184 | - | 730184 | 0.191312 | 6.13803 | 3.759847 | 0.000265 | 0.301401 | 0.040987 |
| 47 | 1570021_at | LOC360030 | HPC14_HUMAN | 360030 | -0.53087 | 4.038423 | -3.75669 | 0.000268 | 0.301401 | 0.032332 |
| 48 | 1558747_at | SMCHD1 | SMHD1_HUMAN | 23347 | -1.11706 | 5.552798 | -3.75163 | 0.000273 | 0.301401 | 0.018436 |
| 49 | 222439_s_at | THRAP3 | Q7Z5U1_HUMAN | 9967 | -0.42358 | 6.33478 | -3.75113 | 0.000274 | 0.301401 | 0.017084 |
| 50 | 235505_s_at | - | - | - | 0.16859 | 4.228702 | 3.744542 | 0.00028 | 0.301401 | -0.00096 |
